# Unusual Enhancement of Doxorubicin Activity on Co-Delivery with Polyhedral Oligomeric Silsesquioxane (POSS)

**DOI:** 10.3390/ma10050559

**Published:** 2017-05-20

**Authors:** Ewelina Sobierajska, Malgorzata Konopka, Anna Janaszewska, Kinga Piorecka, Andrzej Blauz, Barbara Klajnert-Maculewicz, Maciej Stanczyk, Wlodzimierz A. Stanczyk

**Affiliations:** 1 Department of General Biophysics, Faculty of Biology and Environmental Protection, University of Lodz, Pomorska 141/143, 90-236 Lodz, Poland; ewelina-s93@wp.pl (E.S.); gosiapicasa@gmail.com (M.K.); ankuj@poczta.onet.pl (A.J.); aklajn@biol.uni.lodz.pl (B.K.-M.); 2 Centre of Molecular and Macromolecular Studies, Polish Academy of Sciences, Sienkiewicza 112, 90-363 Lodz, Poland; kgradzin@cbmm.lodz.pl; 3 Cytometry Laboratory, Department of Molecular Biophysics, Faculty of Biology and Environmental Protection, University of Lodz, Pomorska 141/143, 90-236 Lodz, Poland; andrzej.blauz@gmail.com; 4 Leibniz-Institut für Polymerforschung Dresden e.V., Hohe Strasse 6, 01069 Dresden, Germany; 5 Copernicus Memorial Hospital, Pabianicka 62, 93-513 Lodz, Poland; macstanczyk@gmail.com

**Keywords:** POSS, silsesquioxane, doxorubicin, co-delivery, in vitro toxicity

## Abstract

Polyhedral oligomeric silsesquioxane (POSS), bearing eight 3-chloroammoniumpropyl substituents, was studied as a potential nanocarrier in co-delivery systems with doxorubicin (DOX). The toxicity of doxorubicin and POSS:DOX complexes at four different molar ratios (1:1; 1:2, 1:4, 1:8) towards microvascular endothelial cells (HMEC-1), breast cancer cells (MCF-7), and human cervical cancer endothelial cells (HeLa) was determined. The rate of penetration of the components into the cells, their cellular localization and the hydrodynamic diameter of the complexes was also determined. A cytotoxicity profile of POSS:DOX complexes indicated that the POSS:DOX system at the molar ratio of 1:8 was more effective than free DOX. Confocal images showed that DOX co-delivery with POSS allowed for more effective penetration of doxorubicin through the cell membrane. Taking all the results into account, it can be claimed that the polyhedral oligomeric silsesquioxane (T_8_-POSS) is a promising, complex nanocarrier for doxorubicin delivery.

## 1. Introduction

Over the last few decades, drug delivery systems have been widely used to treat numerous diseases, cancer in particular. It is believed that such delivery systems offer more advantages when compared to conventional dosage forms, especially by improved drug efficacy and minimized toxicity. Currently, many nanoparticles such as polymers, dendrimers or liposomes are being studied as carriers in drug delivery systems. Although they seem to be promising platforms for cell-specific targeting, there are still major problems that are related to side effects, poor effectiveness and high costs. Thus, the search for superior solutions is important [1,2].

Recently, polyhedral oligomeric silsesquioxanes (POSSs) have attracted significant interest in the biological field owing to their unique structure, nanoscale size (diameters in the range of 1–3 nm) and easy chemical modification [3]. POSSs are nanocages with the empirical formula RSiO_1.5_, consisting of an inorganic silicon and oxygen inner core, as well as an outer shell is made up of organic groups. Examples of such groups range from simple alkyl substituents to functionalized ones, such as methoxyphenyl, 2,4-difluorophenyl [4,5,6,7]. It is believed that silsesquioxanes might be successfully used as targeted carriers of anticancer drugs, however, they have not been intensely explored as POSS-drug conjugates despite their exceptional properties such as nontoxicity and cytocompatibility [8]. The literature related to biomedical applications of POSSs [9] presents data showing that they can be incorporated into polymers and dendrimers, and subsequently conjugated with anticancer drugs via pH sensitive bonds, providing selective drug delivery [10,11]. POSS conjugates with fluorescent dyes were also described as having improved stability that makes them good candidates for cell-imaging [3]. They were used as cardiovascular implants or as agents reducing inflammatory reactions [12]. Recently, the first syntheses of POSS-anthracycline conjugates were reported [13,14].

**Figure 1:**
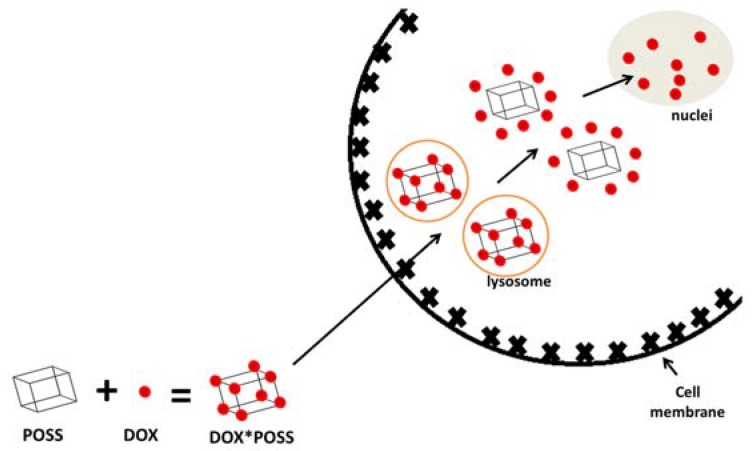
The concept of co-delivery of polyhedral oligomeric silsesquioxane (POSS) and doxorubicin (DOX) into the cell.

Previously, we performed an initial study on the cytotoxicity of POSS-octa(3-chloroammoniumpropyl) silsesquioxane and its impact on mammalian cells. The conclusion drawn from that study was that silsesquioxane was not harmful to cells, what makes it a potential candidate of interest for cell-specific drug delivery [15]. Therefore, we decided to verify the effectiveness of POSS as a carrier of doxorubicin (DOX). In order to do that, we performed a toxicity evaluation of POSS:DOX complexes at four different molar proportions (1:1; 1:2, 1:4, 1:8) towards three cell lines: microvascular endothelial cells (HMEC-1), breast cancer cells (MCF-7), and human cervical cancer endothelial cells (HeLa). Moreover, we checked the rate of uptake of the compounds into the cells, characterized the complexes by measuring their size, and proposed the mechanism explaining the impact of POSS:DOX ratio on the anticancer activity of complexes. Our hypothesis assumes an improvement in transport efficiency of doxorubicin co-delivered with polyhedral oligomeric silsesquioxane into cells (Figure 1).

## 2. Results

The cytotoxicity of DOX and DOX co-delivered with POSS was evaluated by MTT assay in order to compare the influence of these systems at different molar ratios on cell viability of microvascular endothelial (HMEC-1), breast cancer (MCF-7) and human cervical cancer endothelial cells (HeLa). Both doxorubicin and doxorubicin complexes were incubated with cells for 24 and 48 h at various concentrations.

**Figure 2:**
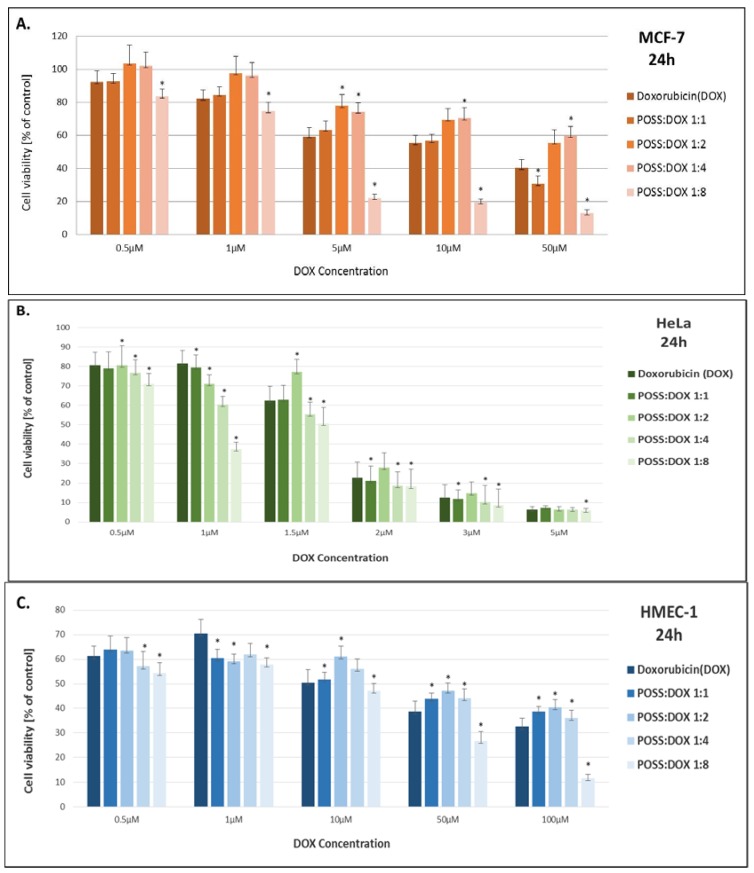
The influence of doxorubicin and polyhedral oligomeric silsesquioxanes co-delivered at different molar ratios on cell viability (**A**) of human breast adenocarcinoma (MCF-7), (**B**) microvascular endothelial (HMEC-1), and (**C**) human cervical cancer endothelial (HeLa) cell lines. Data are presented as a percentage of control ± SD, * *p* < 0.05 (statistical significance between DOX and POSS:DOX complexes).

The cytotoxicity profiles of the compounds incubated with cells for 24 and 48 h exhibited analogous patterns, therefore only the results for 24 h are shown in Figure 2. The complex of POSS:DOX at the ratio of 1:8 demonstrated higher cellular toxicity than free DOX and the other complexes for all of the cell lines. The effect was the most pronounced for the MCF-7 cell line, where statistical differences between free doxorubicin and the complex POSS:DOX at a molar ratio of 1:8 occurred for all concentrations. Interestingly, in a few cases, complexes POSS:DOX at molar ratios of 1:2 and 1:4 showed an opposite effect. The IC_50_ values (Table 1) demonstrate that the complex of polyhedral oligomeric silsesquioxane with doxorubicin at a 1:8 ratio significantly enhances the toxic effect of DOX when compared to a free drug for MCF-7 and HMEC-1 cell lines and, to a smaller extent, for HeLa cells.

Owing to the fact that the possible reason for the enhancement may be the higher cellular uptake of doxorubicin complexed with polyhedral oligomeric silsesquioxane compared to that of free doxorubicin, the rate of penetration of the compounds into the cells was examined using flow cytometry.

**Figure 3:**
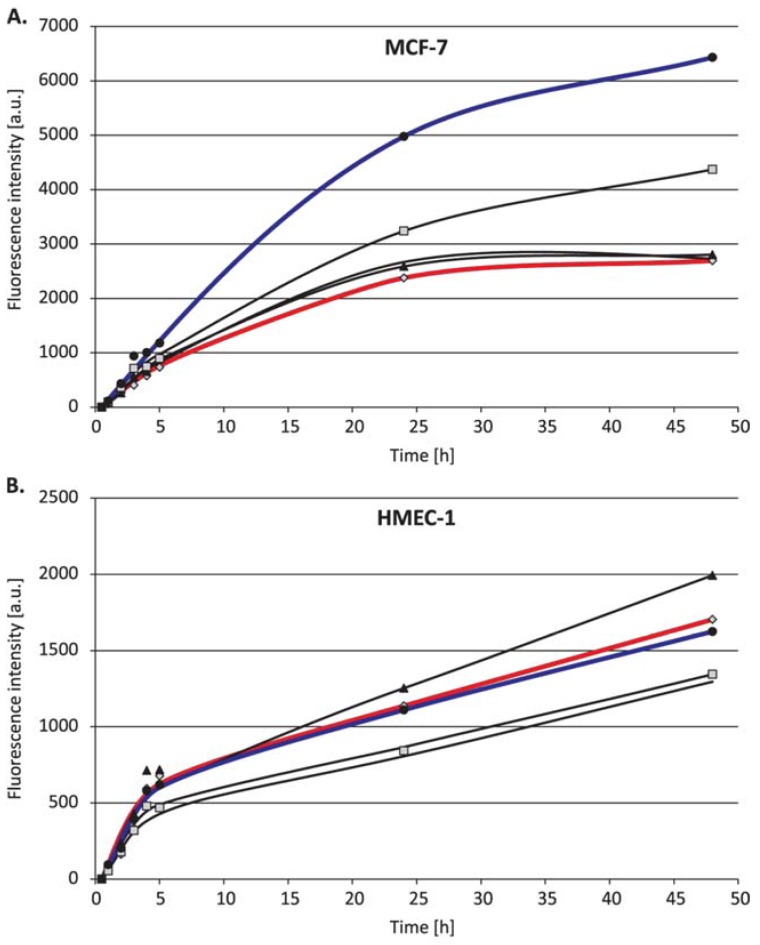

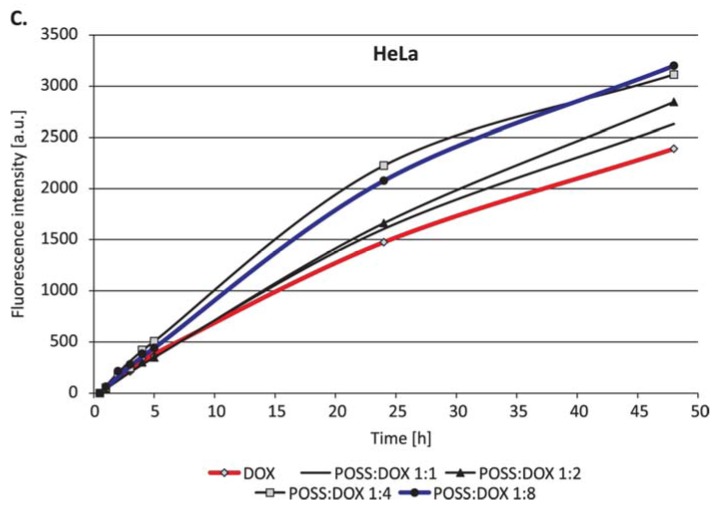
The cellular uptake of doxorubicin (1 µM) and polyhedral oligomeric silsesquioxane co-delivered at different molar ratios by (**A**) human breast adenocarcinoma (MCF-7), (**B**) microvascular endothelial (HMEC-1), and (**C**) human cervical cancer endothelial (HeLa) cell lines.

The flow cytometry results (Figure 3) show the faster internalization of DOX complexed with POSS than that of the free drug, thus confirming that polyhedral oligomeric silsesquioxane contributed to a better penetration of doxorubicin into the human breast adenocarcinoma (MCF-7) and human cervical cancer endothelial (HeLa) cells, which is particularly noticeable for the longer incubation periods (24 and 48 h). In the case of microvascular endothelial cells (HMEC-1), the effect depended on the POSS:DOX ratio.

**Figure 4:**
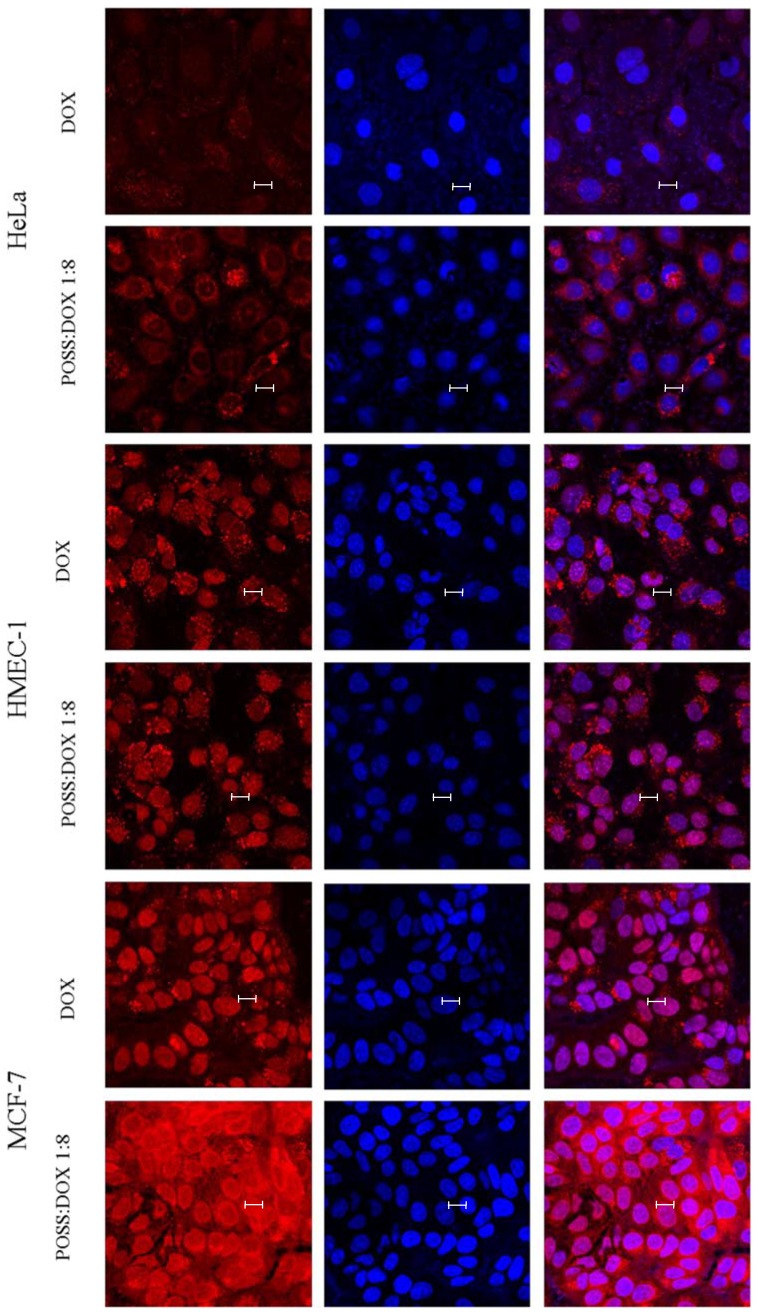
Confocal images of MCF-7, HMEC-1 and HeLa cells treated with 1 µM doxorubicin or doxorubicin (1 µM) co-delivered and polyhedral oligomeric silsesquioxane at POSS:DOX = 1:8 molar ratio. Doxorubicin (red channel), cell nucleus stained with DAPI (blue channel), and overlapping channels. Scale bars: 10 µm.

To verify whether the DOX fluorescence measured by flow cytometry comes from DOX that is localized in the cell or from the surface of the cell, the images visualizing the fluorescence of doxorubicin in the far-red channel and DAPI-stained nuclei in the blue channel were taken, using confocal microscopy for all the three cell lines (Figure 4).

The images of the cells incubated with doxorubicin and POSS:DOX at a 1:8 molar ratio (Figure 4) confirmed our hypothesis that co-delivery allowed for a more effective uptake of doxorubicin through the cell membrane. However, after incorporation into the cell, not all of the drug intercalated directly into DNA; some remained in lysosomes. This may indicate the formation of relatively stable complexes of POSS:DOX. Therefore, the hydrodynamic diameter of potential complexes of doxorubicin with polyhedral oligomeric silsesquioxanes was determined using dynamic light scattering immediately after the addition of the compounds and after 24 h incubation.

**Figure 5:**
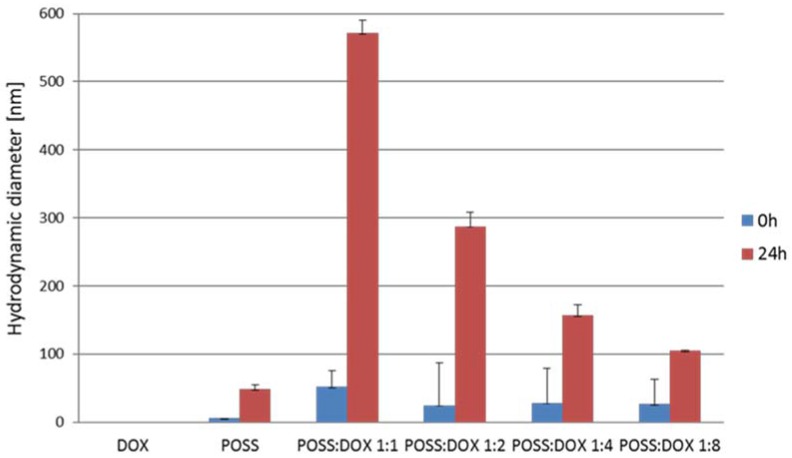
Hydrodynamic diameters of complexes formed by doxorubicin and polyhedral oligomericsilsesquioxanes (50 μM) at different molar ratios. Data are expressed as means ± SD. Statistical differences occurred for POSS and all conjugates at different molar ratios between 0 and 24 h of incubation.

As shown in Figure 5, the diameters measured immediately after the addition of both components indicate that this addition was merely the starting point of the process of forming complexes, and the size of the complexes did not greatly depend on the POSS:DOX molar ratio. This was in agreement with the observation of no significant difference in the cell uptake of complexes at different POSS:DOX ratios when the penetration of the complexes into the cells using a flow cytometer was tested during the first 5 h. However, after 24 h incubation, the hydrodynamic diameter of the formed complexes increased considerably. The difference between the size of the complexes at various molar ratios was statistically significant. With an increase in the concentration of doxorubicin in the sample, the size of complexes decreased, thus confirming the results obtained by the MTT assay, wherein the cytotoxicity of the POSS:DOX complex at a molar ratio of 1:8 was the highest.

## 3. Discussion

**Figure 6:**
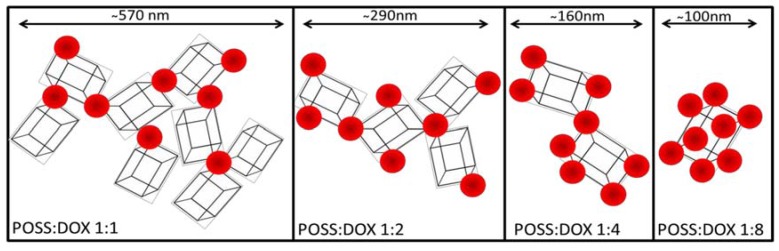
Proposed mechanism of complex formation by doxorubicin and polyhedral oligomeric silsesquioxane at different molar ratios.

Summing up the results obtained from cell viability, cellular uptake, flow cytometry, and measurement of hydrodynamic diameters of the POSS:DOX complexes, we propose a mechanism for the increased activity of doxorubicin-POSS complexes (Figure 6).

This mechanism explains the highest effectiveness of the complex POSS:DOX at a 1:8 molar ratio. We assume that DOX inhibits the self-assembly of oligomeric silsesquioxanes [16,17] and the process is most effective once all functional chlorammoniumpropyl groups of POSS interact with DOX. The size of aggregates determines their interaction with the cell membrane and drug release. These processes lead to changes in the anticancer activity of complexes that substantially depends on the POSS:DOX ratio, as shown in Table 1.

Polyhedral oligomeric silsesquioxanes (POSS) have a wide range of applications in various fields of biomedicine [18,19]. However, there is little data in the literature concerning in vitro studies in terms of their biomedical application. Thus, our study concentrates on the use of these nanocomposites in cancer therapy, which seems to be a good solution to limitations of many anticancer treatments such as drug instability, poor distribution or release in an undesirable location.

Numerous attractive features of this group of compounds distinguish them as unique carriers, such as their nano-size and well-defined shape, thermodynamic stability, biocompatibility, biodegradability, lack of toxicity, high solubility and easy modification. Therapeutics conjugated with POSSs are characterized by selective accumulation in solid tumors and a slower release rate [20]. Our studies show that POSS:DOX at a 1:8 molar ratio demonstrates higher cytotoxicity for cancer cell lines when compared to free doxorubicin. Measurements of the cellular uptake of free doxorubicin and doxorubicin complexed with polyhedral oligomeric silsesquioxanes confirm that POSSs facilitate the penetration of the drug into the cell. The images, obtained from confocal microscopy, indicate that part of doxorubicin which entered the cell is trapped in lysosomes and endosomes, while the rest of the drug intercalates directly into DNA. Measurements of the hydrodynamic diameter of doxorubicin and polyhedral oligomeric silsesquioxane complexes that were taken using dynamic light scattering confirm the formation of stable complexes of POSS:DOX after approximately 5 h, and indicate that the complexes tend to aggregate. The size of the aggregates decreases with a decrease in the POSS:DOX molar ratio and reaches a minimum at a 1:8 molar ratio. The synthesis of the conjugates was described in Reference [21], where doxorubicin was coupled to the nanoglobular surface of generation-3 (G3) poly(l-lysine) dendrimer with octa(3-chlorammoniumpropyl)-silsesquioxane (POSS) as the cubic core via a biodegradable disulfide spacer. Subsequently, the authors estimated the cytotoxicity in U87 glioblastoma cells overexpressing avb3 integrin. The conjugate showed a higher cytotoxicity than free doxorubicin at the higher drug concentration (around 65 mg/mL) after 48 h incubation. The results from our studies are in accordance with these data. Another similarity to this previous research is the fact that some of the compounds that entered the cell were trapped in lysosomes and endosomes. Other researchers [22] carried out experiments with POSSs bearing the same octa(3-chlorammoniumpropyl) substituents as the core of poly(l-glutamic acid) dendrimers that are linked with doxorubicin via a hydrazone bond. They also observed that doxorubicin was released in an acid intracellular environment (lysosomes or endosomes).

The current work has shown that the simple co-delivery of doxorubicin with polyhedral oligomeric silsesquioxane (POSS) bearing eight 3-chloroammoniumpropyl substituents significantly enhances the activity of the anthracycline. We studied the activity of doxorubicin and POSS:DOX complexes towards microvascular endothelial cells (HMEC-1), breast cancer cells (MCF-7), and human cervical cancer endothelial cells (HeLa), and our data pointed to 1.5–6-fold increase in toxicity. The rate of penetration of the components into the cells, their cellular localization, and their hydrodynamic diameter allowed for a presentation of the most probable mechanism of action for such complexes. A cytotoxicity profile of POSS:DOX complexes indicated that conjugate POSS:DOX at a molar ratio of 1:8 was more effective than free DOX. Thus, it appears that POSS can be regarded as an interesting candidate nanocarrier for doxorubicin delivery, though further studies are needed to explore the characteristics of the complexes and finally prove the mechanism of their action.

## 4. Materials and Methods

### 4.1. Materials

POSS was made as described in Reference [23] from (3-aminopropyl) triethoxysilane (Sigma Aldrich, Poznan, Poland). All cell culture reagents were purchased from Gibco (Darmstadt, Germany). Flasks and multi-well plates were obtained from Nunc (Darmstadt, Germany). MTT (3-[4,5-dimethylthiazol-2-yl]-2,5-diphenyltetrazolium bromide) and doxorubicin were obtained from Sigma Aldrich Company (Poznan, Poland). Microvascular endothelial cell line (HMEC-1), human breast adenocarcinoma cell line (MCF-7), and human cervical cancer endothelial cell line (HeLa) were obtained from American Type Culture Collection (ATCC).

### 4.2. Cell Cultures

Microvascular Endothelial Cells (HMEC-1) were grown in an MCDB131 medium supplemented with l-glutamine, hydrocortisone and epidermal growth factor. Breast cancer cells (MCF-7) were grown in a MEM (Minimum Essential Media) medium, while human cervical cancer endothelial cells (HeLa) were grown in DMEM (Dulbecco's Modified Eagle Medium) medium. 10% fetal bovine serum (FBS) and streptomycin (100 mg/mL) were added to all cell culture media. The cells were grown in T-75 culture flasks at 37 °C in an atmosphere containing 5% CO_2_. HMEC-1 and HeLa were sub-cultured every 2 days, while MCF was sub-cultured every 3 days. The viability of cells was determined by the trypan blue exclusion assay with the use of a Countess Automated Cell Counter (Invitrogen, Carlsbad, CA, USA). Cells were seeded either in 96-well plates at a density of 1.0 × 10^4^ cells/well in 100 μL of an appropriate medium (HeLa), or at a density of 2.0 × 10^4^ cells/well in 100 μL of an appropriate medium (HMEC-1 and MCF-7), or in 12-well plates at a density of 1.0 × 10^5^ cells/well and 2.0 × 10^5^ cells/well in 1 mL of an appropriate medium, respectively. After seeding, the plates were incubated for 24 h in a humidified atmosphere containing 5.0% CO_2_ at 37 °C in order to allow cells to attach to the plates.

### 4.3. In Vitro Toxicity 

The cytotoxicity of POSS, doxorubicin (DOX) and co-delivered POSS/doxorubicin (at molar ratios of 1:1, 1:2, 1:4, and 1:8) was determined by MTT assay [24]. The cells were seeded on 96-well plates at a density of 2.0 × 10^4^ cells/well (HMEC-1 and MCF-7) and 1.0 × 10^4^ cells/well (HeLa). The compounds at a concentration range from 1 to 100 µM were added. Cells were incubated for 24 and 48 h. After incubation, the medium was removed and cells were washed with PBS. Next, 50 µL of 0.5 mg/mL MTT solution in PBS was added to each well and cells were further incubated under normal culture conditions for 4 h. After incubation, the MTT solution was removed and the obtained formazan precipitate was dissolved in dimethyl sulfoxide (DMSO) (100 µL/well). The conversion of the tetrazolium salt (MTT) to a colored formazan by mitochondrial and cytosolic dehydrogenases was a marker of cell viability. Before the absorbance measurement, plates were shaken for 1 min and the absorbance at 570 nm was measured with a PowerWave HT Microplate Spectrophotometer (BioTek, Winooski, VT, USA).

### 4.4. Uptake Detection

In vitro uptake studies were carried out using fluorescent doxorubicin and co-delivered POSS/doxorubicin (at molar ratios of 1:1, 1:2, 1:4, and 1:8). The compounds were added at a concentration of 1 µM doxorubicin to the 12-well plates containing cells at a density of 1.0 × 10^5^ cells/well (HeLa) or 2.0 × 10^4^ cells/well (HMEC-1 and MCF-7). In this study, cells were incubated with the compounds for a specific period of time in a range from 5 min to 48 h in a humidified atmosphere containing 5.0% CO_2_ at 37 °C. After the appropriate incubation period, cells were washed with PBS, suspended in 500 µL of medium and immediately analyzed with a Becton Dickinson LSR II flow cytometer (BD Biosciences, San Jose, CA, USA) using a blue laser at 488 nm and PE (phycoerthrin) band pass filter BD, Franklin Lakes, NJ, USA at 575/26 nm.

### 4.5. Confocal Microscopy

Confocal microscopy images were obtained under 6300× magnification with a Zeiss LSM 780 microscope equipped with a 405-nm laser diode and an In Tune excitation laser system (Carl Zeiss Micro Imaging, Thornwood, NY, USA). Cells were grown on 96-well glass-bottom plates and incubated with 1 µM doxorubicin or co-delivered POSS/doxorubicin (at molar ratios of 1:1, 1:2, 1:4, and 1:8) for 24 h at 37 °C in a humidified atmosphere containing 5.0% CO_2_. After incubation, cells were cooled on ice and washed once with cold phosphate buffered saline (PBS) to inhibit endocytosis. Cell nuclei were stained with DAPI in PBS for 10 min and fixed with 3.6% formaldehyde solution for 15 min at room temperature. Fixed and stained cells were imaged to visualize the fluorescence of doxorubicin in the far-red channel (excitation 595 nm, emission 600–740 nm) and nuclei in the blue channel (excitation 405 nm, emission 410–470 nm).

### 4.6. Hydrodynamic Diameter

Hydrodynamic diameters of particles were measured in deionized water at 37 °C by dynamic light scattering (DLS) using Zetasizer Nano—ZS from Malvern (Malvern, UK). DLS measures Brownian motions and relates this to the size of particles by illuminating the particles with a laser light (633 nm) and analyzing the intensity fluctuations in scattered light.

Hydrodynamic diameter measurements were made for pure POSS (50 μM) and for complexes formed by doxorubicin with POSS at different molar ratios (1:1; 2:1; 4:1; 8:1) immediately after mixing the components and after 24 h incubation. For each sample, measurements were performed three times in three replications.

### 4.7. Statistical Analysis

Data were expressed as means ± SD. Analysis of variance (ANOVA) with post hoc test (Tukey) was used for multiple comparisons. Statistics were calculated using Statistica software (StatSoft, Tulsa, OK, USA), and *p*-values < 0.05 were considered significant.

## Figures and Tables

**Table 1:** Comparison of IC_50_ values for free doxorubicin and doxorubicin co-delivered with polyhedral oligomeric silsesquioxane in three different cell lines.

Sample	IC_50_ [µM/L]		
	MCF-7	HeLa	HMEC-1
Doxorubicin (DOX)	17.44 ± 5.23	1.45 ± 0.15	10.33 ±4.63
POSS:DOX 1:1	13.65 ± 4.03	1.44 ± 0.12	10.92 ± 2.26
POSS:DOX 1:2	76.97 ± 35.11	1.61 ± 0.26	14.81 ± 12.10
POSS:DOX 1:4	109.10 ± 55.88	1.25 ± 0.08	9.11 ± 4.56
POSS:DOX 1:8	2.69 ± 0.15	0.92 ± 0.09	2.51 ± 0.42
